# Adherence and Persistence with Once-Daily Teriparatide in Japan: A Retrospective, Prescription Database, Cohort Study

**DOI:** 10.1155/2013/654218

**Published:** 2013-12-12

**Authors:** Ikuko Tanaka, Masayo Sato, Tomoko Sugihara, Douglas E. Faries, Shuko Nojiri, Peita Graham-Clarke, Jennifer A. Flynn, Russel T. Burge

**Affiliations:** ^1^Nagoya Rheumatology Clinic/Initiative for Rheumatology and Osteoporosis, Nagoya 4500002, Japan; ^2^Lilly Research Laboratories Japan, Eli Lilly Japan K.K., 7-1-5 Isogami dori, Chuoku, Kobe 6510086, Japan; ^3^Statistical Programming, PharmaNet/i3 LLC, Indianapolis, IN 46280, USA; ^4^Statistics, Eli Lilly and Company, Indianapolis, IN 46285, USA; ^5^Surveillance & Epidemiology, Global Patient Safety Japan, Eli Lilly Japan K.K., Kobe 6510086, Japan; ^6^Global Health Outcomes, Eli Lilly Australia Pty Ltd, Sydney 2114, Australia; ^7^Global Health Outcomes, Eli Lilly and Company, Indianapolis, IN 46285, USA

## Abstract

Adherence and persistence with osteoporosis treatments are essential for reducing fracture risk. Once-daily teriparatide is available in Japan for treating osteoporosis in patients with a high risk of fracture. The study objective was to describe real-world adherence and persistence with once-daily teriparatide 20 *μ*g during the first year of treatment for patients who started treatment during the first eight months of availability in Japan. This prescription database study involved patients with an index date (first claim) between October 2010 and May 2011, a preindex period ≥6 months, and a postindex period ≥12 months and who were aged >45 years. Adherence (medication possession ratio (MPR)) and persistence (time from the start of treatment to discontinuation; a 60-day gap in supply) were calculated. A total of 287 patients started treatment during the specified time period; 123 (42.9%) were eligible for inclusion. Overall mean (standard deviation) adherence was 0.702 (0.366), with 61.0% of patients having high adherence (MPR > 0.8). The percentage of patients remaining on treatment was 65.9% at 180 days and 61.0% at 365 days. Our findings suggest that real-world adherence and persistence with once-daily teriparatide in Japan are similar to that with once-daily teriparatide in other countries and with other osteoporosis medications.

## 1. Introduction

Osteoporosis is a very common disease in Japan that is associated with significant morbidity, mortality, and economic/social burdens [1–7]. Indeed, recent estimates from Japan suggest that 3.4%–12.4% of men and 16.3%–26.5% of women (mean (standard deviation) age: 69.9 (11.2) years) have lumbar, femoral neck, or hip osteoporosis [1]. If left untreated, osteoporosis can lead to osteoporotic fractures and increased mortality [2–5]. A number of factors increase the risk of subsequent fractures, including advanced age, low bone mineral density (BMD), previous vertebral fracture, and previous clinical fracture [8]. The number of osteoporotic fractures in Japan has increased during the last 20 years as the elderly population has increased in number [2, 3, 9]. As the size of the elderly population is expected to continue increasing in the future, the prevalence of osteoporosis in Japan will also increase [1]. Therefore, determining the most effective osteoporosis treatments is essential [1].

Long-term adherence and persistence with medications in the treatment of chronic conditions are crucial for preventing increases in morbidity, mortality, and healthcare costs [10]. In particular, long-term adherence and persistence are important factors underlying the effectiveness of osteoporosis treatments [11, 12]; both are essential for increasing BMD and reducing the risk of fracture [13]. Common reasons for poor adherence and persistence include adverse events, inconvenience of treatment, and the lack of appropriate patient education [13]. Poor adherence can have serious health consequences, including smaller increases in BMD and an increased risk of new fractures [14]. Indeed, there is an inverse relationship between treatment adherence and fracture probability [15–18]. Poor adherence also increases general healthcare costs and decreases the cost-effectiveness of treatment [19, 20].

Adherence and persistence with osteoporosis treatments in clinical practice are generally poor [21–23], with approximately 50% of patients not adhering or persisting with treatment after 12 months [24]. Further, a meta-analysis of observational studies found that only 53% of patients achieved a medication possession ratio (MPR) ≥ 0.8 six months after starting treatment and that only 43% of patients achieved an MPR ≥ 0.8 7–12 months after starting treatment [25]. There are very limited data in the peer-reviewed literature on adherence and persistence with osteoporosis medications in Japan [26–28]. Findings from the only real-world clinical practice study in Japan published to date suggest that adherence with osteoporosis medications may be low, with approximately 45% of patients found to be adherent with bisphosphonates 12 months after starting treatment [26].

Once-daily teriparatide, the recombinant 1–34 fragment of human parathyroid hormone, became available in Japan in October 2010 for the treatment of osteoporosis in patients with a high risk of fracture. Given as a subcutaneous injection, teriparatide stimulates osteoblastic activity and the formation of new trabecular and cortical bone [29]. Clinical studies have shown that once-daily teriparatide administered over 18–24 months significantly reduces the incidence of vertebral, nonvertebral, and fragility fractures, reduces back pain, and improves quality of life in postmenopausal women with osteoporosis [30, 31]. Adherence and/or persistence with once-daily teriparatide have been investigated in studies carried out in Europe and North America [18, 31–36]. After 12 months, adherence (MPR) in these studies ranged from 0.66–0.81 [18, 34], whereas persistence ranged from 57%–87% [18, 31, 32, 34, 35]. It is unclear whether once-daily teriparatide adherence and persistence patterns in Japan are similar to those in other countries or to those for other osteoporosis treatments.

The aim of this prescription database study was to describe real-world adherence and persistence with once-daily teriparatide during the first year of treatment for patients who started treatment during the first eight months of availability in Japan.

## 2. Materials and Methods

### 2.1. Data Source

Data were obtained from a pharmacy prescription database provided by Japan Medical Information Research Institute Inc. (Tokyo, Japan). The database comprises the deidentified, pharmacy-specific records of approximately 630,000 patients, 840,000 prescriptions, and 45,000 prescribing physicians per month. Further, the database includes 0.8% (400/50,000) of pharmacies and 1.5 to 2.0% of prescriptions nationwide and contains information on demographics, dispensing records, prescribers, hospital size, and type of insurance. The age, sex, and prescriber distributions in the database are consistent with those of the general Japanese population (Japan Medical Information Research Institute Inc., Tokyo, Japan).

### 2.2. Study Population

Men and women were eligible for inclusion if they had an index date (first claim for once-daily teriparatide 20 *μ*g (Forteo, rhPTH(1-34), Eli Lilly Japan K.K., Kobe, Japan)) between October 2010 and May 2011, a preindex period ≥6 months, a postindex period ≥12 months, and aged >45 years.

Demographic and clinical information were recorded at the index date. Medications used during the pre-index period were recorded.

### 2.3. Study Objectives

The primary study objectives were to assess adherence and persistence during the first 12 months of treatment.

Adherence was measured by calculating the MPR, defined as the sum of the days' supply of medication dispensed between the start and the end of the study period divided by the total number of days in the study period. Once-daily teriparatide is prescribed in 30-day increments (note that once-daily teriparatide received an exemption from the 14-day supply limit that is standard during the first 12 months after approval for any new medication in Japan). Therefore, the days' supply of medication for most prescriptions fell into one of three increments: 30 (one month), 60 (two months), or 90 days (three months).

Persistence was calculated using the time from the start of treatment to the discontinuation of treatment or the end of the study period. Discontinuation was defined as a 60-day gap between two prescriptions or discontinued use of once-daily teriparatide.

Secondary objectives were to compare baseline demographic and clinical characteristics between patients who had and had not been prescribed glucocorticoids during the pre-index period; to compare baseline demographic and clinical characteristics, adherence, and persistence between patients who were early (months 1–4 of availability) and later (months 5–8 of availability) initiators of treatment; and to assess the proportion of patients switching treatment/restarting treatment after discontinuation. We compared characteristics between patients who had and had not been prescribed glucocorticoids because glucocorticoid use is an established risk factor for osteoporosis [37]. We compared characteristics, adherence, and persistence between patients who were early and later initiators of once-daily teriparatide to explore potential differences (e.g., due to patient education and prescribing physicians' attitudes/knowledge) related to the timing of approval in Japan.

### 2.4. Statistical Analysis

Demographic and clinical characteristics were summarised for the full analysis sample and were compared (two-sample *t*-test for continuous variables/Fisher's exact test for categorical variables) between patients who had and had not been prescribed glucocorticoids during the pre-index period and between patients who were early and later initiators of treatment.

Variables associated with high adherence (MPR > 0.80) were identified using stepwise logistic regression (odds ratio (OR) and 95% confidence interval (CI)), with age, sex, timing of treatment initiation, prescribing physician's specialty, hospital size, insurance type, and previous medications as potential covariates. The cut-off for high adherence was selected in accordance with previous studies reporting adherence with osteoporosis medications [16, 18]. A Kaplan-Meier survival curve for persistence was plotted to summarise the discontinuation trend for once-daily teriparatide. Variables associated with persistence were identified using a stepwise Cox proportional hazard model (hazards ratio (HR) and 95% CI), with age, sex, timing of treatment initiation, prescribing physician's specialty, hospital size, insurance type, and previous medications as potential covariates. Adherence and persistence for patients who were early and later initiators of treatment were analysed as described above.

Differences were considered statistically significant if *P* < 0.05. No adjustments for multiplicity were performed. Data were analysed using SAS software, version 9.2 (SAS Institute Inc., Cary, NC).

## 3. Results

**Figure 1:**
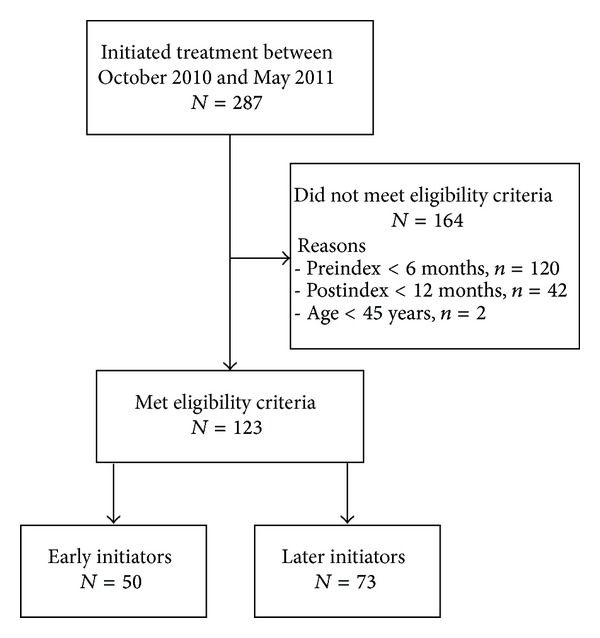
Patient disposition. Early initiators started treatment during months 1–4 of once-daily teriparatide availability, whereas later initiators started treatment during months 5–8 of once-daily teriparatide availability.

A total of 287 patients in the database initiated treatment between October 2010 and May 2011. Of these patients, 123 (42.9%) met the eligibility criteria and 164 (57.1%) did not (Figure 1).

### 3.1. Patient Characteristics

Of the 123 patients included in the study, the majority (≥70%) were aged ≥70 years, were women, and were prescribed once-daily teriparatide by physicians who were orthopaedic specialists (Table 1). Two-thirds of patients had been prescribed osteoporosis medications during the pre-index period, most commonly bisphosphonates. Other common (≥40% of patients) disease prescriptions during the pre-index period included medications for rheumatoid arthritis and cardiovascular disease. No patients were aged between 45 and 54 years.

There were several statistically significant differences in demographic and clinical characteristics between patients who were and were not prescribed glucocorticoids during the pre-index period and between patients who were early and later initiators of treatment (Table 1). Compared with patients who were not prescribed glucocorticoids, patients who were prescribed glucocorticoids were younger, were more commonly prescribed medications for osteoporosis, rheumatoid arthritis, and cardiovascular disease, and were more commonly prescribed immunosuppressants and anticoagulants during the pre-index period (*P* < 0.05). Compared with patients who were early initiators of once-daily teriparatide, patients who were later initiators of once-daily teriparatide were less commonly prescribed raloxifene, vitamin D, and calcium and more commonly prescribed medications for rheumatoid arthritis during the pre-index period (*P* < 0.05).

### 3.2. Adherence

Overall mean (standard deviation) MPR was 0.702 (0.366), with 61.0% of patients having high adherence (MPR > 0.8) (Table 2). Adherence (mean and MPR > 0.8) was numerically, but not statistically significantly, higher for patients who were later initiators of treatment compared with patients who were early initiators of treatment (Table 2). Adherence was similar between patients who were and were not prescribed glucocorticoids during the pre-index period (data not shown).

The odds of high adherence were significantly greater for patients aged 70–79 years than those aged ≥80 years (OR: 4.001; 95% CI: 1.533–10.445; *P* = 0.014) and for women than for men (OR: 6.377; 95% CI: 1.233–32.980; *P* = 0.027).

### 3.3. Persistence

**Figure 2:**
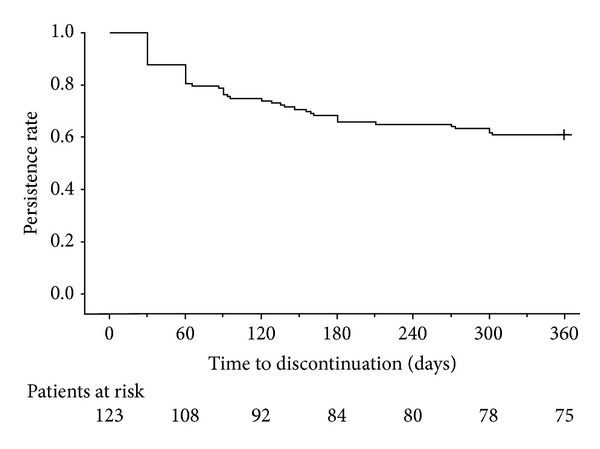
Survival curve for overall persistence with once-daily teriparatide during the 12-month study period. Persistence was defined as the time from the start of treatment to the discontinuation of treatment. Discontinuation was defined as a 60-day gap in once-daily teriparatide supply. + indicates censored.

**Figure 3:**
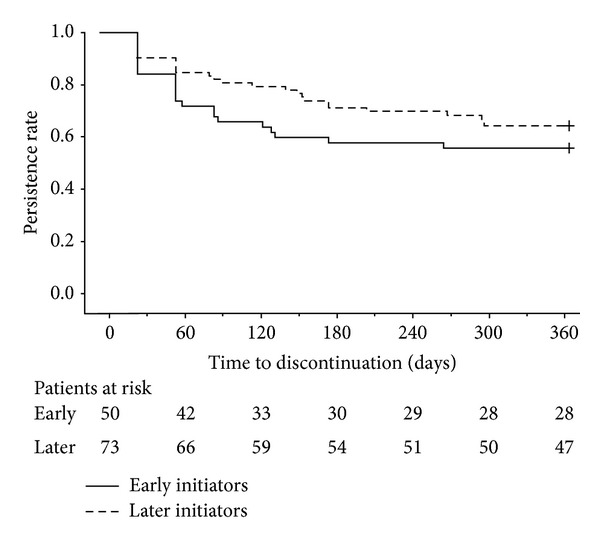
Survival curve for persistence with once-daily teriparatide during the 12-month study period by time frame of treatment. Early initiators started treatment during months 1–4 of once-daily teriparatide availability, whereas later initiators started treatment during months 5–8 of once-daily teriparatide availability. Persistence was defined as the time from the start of treatment to the discontinuation of treatment. Discontinuation was defined as a 60-day gap in once-daily teriparatide supply. + indicates censored.

The percentage of patients remaining on treatment was 65.9% at 180 days and 61.0% at 365 days (Table 2, Figure 2). Persistence with treatment was numerically, but not statistically significantly, higher for patients who were later initiators of treatment compared with patients who were early initiators of treatment (Table 2, Figure 3). The decrease in persistence was most evident within the first 60 days, particularly for patients who were early initiators (Figure 3). Persistence was similar between patients who were and were not prescribed glucocorticoids during the pre-index period (data not shown).

The likelihood of discontinuation was significantly lower for women than men (HR: 0.404; 95% CI: 0.179–0.910; *P* = 0.029) and for patients prescribed rheumatoid arthritis medications during the pre-index period (HR: 0.535; 95% CI: 0.290–0.989; *P* = 0.046) and was significantly higher for patients prescribed calcium (HR: 2.588; 95% CI: 1.283–5.222; *P* = 0.008) or anticonvulsants (HR: 6.510; 95% CI: 1.829–23.169; *P* = 0.004) during the pre-index period.

### 3.4. Proportion of Patients Switching/Restarting Treatment

Of the 123 patients, 27 (22.0%) switched to other osteoporosis medications and 6 (4.9%) restarted once-daily teriparatide treatment after discontinuation. The most common osteoporosis medications switched to were bisphosphonates (7 patients; 25.9%) and alfacalcidol + calcium (6 patients; 22.2%).

## 4. Discussion

In this study, we evaluated real-world adherence and persistence with once-daily teriparatide in Japan. We extracted data from a pharmacy claims database and determined 12-month rates of adherence and persistence with once-daily teriparatide for patients who started treatment during the first eight months of availability. Given the importance of adherence and persistence for maintaining the effectiveness of osteoporosis treatments and that once-daily teriparatide has only recently become available in Japan, our findings represent timely information on the clinical use of once-daily teriparatide in Japan.

In general, the estimates for once-daily teriparatide adherence and persistence in our study are consistent with those from similar studies conducted in other countries. Using United States prescription databases, Foster et al. [34] and Yu et al. [18] reported mean MPRs for once-daily teriparatide at 12 months of 0.66 and 0.81 and persistence rates of 56.7% and 69.0%. The most likely explanation for the higher MPR and rate of persistence in the Yu et al. study is that patients were excluded from the study if they had only one prescription for once-daily teriparatide. This contrasts with our study and the Foster et al. study [34], where all patients with at least one prescription for once-daily teriparatide were included. The use of prescription data to estimate adherence does not allow for confirmation of medication administration and, therefore, may overestimate adherence. Despite this, several nonprescription database studies carried out in Canada [38] and Europe [31, 35] have reported higher estimates for adherence and persistence with once-daily teriparatide than in our study. However, these studies involved patients with severe disease [31, 35] and/or patient-reported assessments of adherence and persistence [31, 35, 38], which may have contributed to the higher estimates compared with our own.

The estimates of adherence and persistence with once-daily teriparatide in our study compare favourably with those for other osteoporosis medications. Of note, our estimates of adherence and persistence are not greatly different to those from a prescription database study of once-daily alendronate (12-month MPR: 0.576; persistence: 31.7%) [39], a claims database study of osteoporosis medications (medication days <80% of possible: 70% of patients at 12 months; persistence: 53%) [40], and a meta-analysis of observational studies examining real-world adherence with osteoporosis medications (MPR ≥0.8 at 7–12 months: 43%; persistence: 50%) [25]. Our estimates of adherence compare favourably with those from a clinical practice study of Japanese women with osteoporosis, in which approximately 45% of patients were adherent with bisphosphonates at 12 months [26]. Overall, the available evidence suggests that treatment with once-daily teriparatide is not associated with decreases in adherence and persistence relative to other osteoporosis medications. This is an important point because teriparatide is administered as a once-daily, self-administered subcutaneous injection, which is unlike most other osteoporosis medications that are taken orally and may require less frequent dosing.

We found that several factors were significantly associated with high adherence and persistence. However, these results are limited by the small number of patients and because we did not have information on a number of other factors that may have affected adherence and persistence, such as disease severity, fracture history, or adverse events. As adherence and persistence are critical for osteoporosis treatments to be effective [11, 12], identifying reliable predictors of adherence and persistence with once-daily teriparatide in Japan may help provide information on how to optimise treatment strategies. Hence, more robust analyses on factors affecting adherence and persistence with once-daily teriparatide in Japan are warranted as additional data become available.

Subgroup analysis of the data in our study suggests that later initiators of treatment had higher rates of adherence and persistence than early initiators of treatment. One potential explanation for this finding is the support program for patients taking once-daily teriparatide. Indeed, the importance of patient support/education for persistence with teriparatide has been demonstrated in studies carried out in Europe [32, 33]. In Japan, the support program for patients taking once-daily teriparatide aims to educate and train patients on the use of once-daily teriparatide to improve adherence and persistence. Although we did not assess the effectiveness of the support program in this study, we suggest that the teriparatide support program may have been less well established in the earlier part of the study, which commenced shortly after once-daily teriparatide became available. Therefore, patients who were early initiators of treatment may have received less comprehensive education/training than patients who were later initiators of treatment. Interestingly, we found that the decrease in persistence was most pronounced shortly after starting treatment. This finding further emphasises the importance of early and effective patient education to help maximise adherence and persistence. Adherence can also be affected by physicians' attitudes and knowledge [23]. We suggest that this may be particularly true for newly approved treatments. Therefore, another potential explanation for the different rates of adherence and persistence between early and later initiators of treatment is differing physician attitudes and knowledge about once-daily teriparatide. For example, physicians treating patients who were early initiators of treatment may have been less knowledgeable about teriparatide than physicians treating patients who were later initiators of treatment.

The strengths of our study are that the data were obtained from clinical practice settings within a short time after once-daily teriparatide became available in Japan. Indeed, our study highlights the benefits of using prescription databases to rapidly obtain and analyse data on drug use in clinical settings. However, the small number of patients in our study and the lack of available information on additional patient variables (e.g., disease diagnosis and severity, comorbidities, fracture history, fractures during treatment, adverse events, hospitalisation, functioning, treatment costs, and reasons for changing medication/treatment discontinuation) limited our capacity to produce definitive statistical conclusions regarding the association between these variables and the rates of adherence and persistence in this patient population. Similar prescription database studies carried out in the United States have examined some of these associations. Specifically, Foster et al. [34] reported that BMD screening, use of antiresorptive therapies within 12 months of starting teriparatide, and lower treatment costs were associated with better persistence with teriparatide. Yu et al. [18] reported that risk of fracture was lower in patients with high adherence (MPR ≥ 0.80) and that persistence with teriparatide for 19 to 24 months was associated with a lower risk of vertebral and nonvertebral fractures compared with persistence for 1 to 6 months. An additional limitation of our study is that, because once-daily teriparatide has only recently become available in Japan, the length of follow-up was relatively short given that the treatment regimen is 24 months. Hence, a study with a greater length of follow-up and a larger number of patients is warranted.

## 5. Conclusion

In conclusion, our findings suggest that adherence and persistence with once-daily teriparatide in Japan, as determined using prescription data for patients who started treatment during the first eight months of availability, are similar to adherence and persistence with once-daily teriparatide in other countries. Despite requiring once-daily injection, adherence and persistence with teriparatide appear to be similar to adherence and persistence with other osteoporosis medications, including oral medications with less frequent dosing.

## Figures and Tables

**Table 1:** Characteristics of patients who had prescriptions for once-daily teriparatide: overall, by glucocorticoid prescription, and by time frame.

Characteristic	All (*N* = 123) *n* (%)	Glucocorticoids^a^			Time frame of treatment^b^		
		No (*N* = 97) *n* (%)	Yes (*N* = 26) *n* (%)	*P* value	Early (*N* = 50) *n* (%)	Later (*N* = 73) *n* (%)	*P* value
Age (years)				0.002			0.972
55–69	36 (29.3)	21 (21.6)	15 (57.7)		14 (28.0)	22 (30.1)	
70–79	52 (42.3)	44 (45.4)	8 (30.8)		22 (44.0)	30 (41.1)	
≥80	35 (28.5)	32 (33.0)	3 (11.5)		14 (28.0)	21 (28.8)	
Sex				1.000			0.526
Women	113 (91.9)	89 (91.8)	24 (92.3)		45 (90.0)	68 (93.2)	
Men	10 (8.1)	8 (8.2)	2 (7.7)		5 (10.0)	5 (6.8)	
Insurance type				0.858			0.246
Late-stage elderly healthcare	71 (57.7)	57 (58.8)	14 (53.8)		33 (66.0)	38 (52.1)	
National health insurance	36 (29.3)	28 (28.9)	8 (30.8)		13 (26.0)	23 (31.5)	
Other	16 (13.0)	12 (12.4)	4 (15.4)		4 (8.0)	12 (16.4)	
Hospital size				0.008			0.002
<200 beds	55 (44.7)	50 (51.5)	5 (19.2)		30 (60.0)	25 (34.2)	
200–499 beds	41 (33.3)	29 (29.9)	12 (46.2)		16 (32.0)	25 (34.2)	
≥500 beds	27 (22.0)	18 (18.6)	9 (34.6)		4 (8.0)	23 (31.5)	
Physician speciality				<0.001			0.148
Orthopaedics	109 (88.6)	93 (95.9)	16 (61.5)		41 (82.0)	68 (93.2)	
Rheumatology	9 (7.3)	1 (1.0)	8 (30.8)		6 (12.0)	3 (4.1)	
Other	5 (4.1)	3 (3.1)	2 (7.7)		3 (6.0)	2 (2.7)	
Osteoporosis medication (any)	82 (66.7)	59 (60.8)	23 (88.5)	0.009	37 (74.0)	45 (61.6)	0.176
Bisphosphonates^c^	69 (56.1)	49 (50.5)	20 (76.9)	0.025	31 (62.0)	38 (52.1)	0.355
Raloxifene	23 (18.7)	16 (16.5)	7 (26.9)	0.260	15 (30.0)	8 (11.0)	0.010
Ipriflavone	1 (0.8)	1 (1.0)	0 (0.0)	1.000	1 (2.0)	0 (0.0)	0.407
Disease-related medications							
Vitamin D	50 (40.7)	36 (37.1)	14 (53.8)	0.177	26 (52.0)	24 (32.9)	0.041
Calcium	20 (16.3)	15 (15.5)	5 (19.2)	0.765	13 (26.0)	7 (9.6)	0.024
Immunosuppressants	13 (10.6)	4 (4.1)	9 (34.6)	<0.001	4 (8.0)	9 (12.3)	0.557
Anticonvulsants	4 (3.3)	2 (2.1)	2 (7.7)	0.196	2 (4.0)	2 (2.7)	1.000
Major disease prescriptions							
Rheumatoid arthritis	60 (48.8)	39 (40.2)	21 (80.8)	<0.001	18 (36.0)	42 (57.5)	0.027
Cardiovascular disease	51 (41.5)	35 (36.1)	16 (61.5)	0.025	20 (40.0)	31 (42.5)	0.853
Anticoagulants	29 (23.6)	18 (18.6)	11 (42.3)	0.018	16 (32.0)	13 (17.8)	0.085
Central nervous system	26 (21.1)	19 (19.6)	7 (26.9)	0.425	9 (18.0)	17 (23.3)	0.510
COPD	10 (8.1)	7 (7.2)	3 (11.5)	0.439	3 (6.0)	7 (9.6)	0.739
Oral antidiabetics or insulin	4 (3.3)	2 (2.1)	2 (7.7)	0.196	1 (2.0)	3 (4.1)	0.645
Parkinson's disease	4 (3.3)	3 (3.1)	1 (3.8)	1.000	2 (4.0)	2 (2.7)	1.000

COPD: chronic obstructive pulmonary disease.

Data were compared by two-sample *t*-test or Fisher's exact test.

^a^Prescription of glucocorticoids during the six months before the first claim for once-daily teriparatide.

^b^Early initiators started treatment during months 1–4 of once-daily teriparatide availability, whereas later initiators started treatment during months 5–8 of once-daily teriparatide availability.

^c^Includes alendronate, etidronate, minodronate, and risedronate.

*P* < 0.05 indicates statistical significance.

**Table 2:** Adherence and persistence with once-daily teriparatide during the 12-month study period.

Variable	All (*N* = 123)	Time frame of treatment^a^	
		Early (*N* = 50)	Later (*N* = 73)
*Adherence *			
MPR^b^, mean (SD)	0.702 (0.366)	0.640 (0.398)	0.745 (0.339)
MPR > 0.8, *n* (%)	75 (61.0)	27 (54.0)	48 (65.8)
*Persistence *			
Estimated TTD^c^, days			
25th percentile	95	60	161
50th percentile	Censored (≥365)	Censored (≥365)	Censored (≥365)
75th percentile	Censored (≥365)	Censored (≥365)	Censored (≥365)
Actual TTD, *n* (%)			
>0 days	123 (100.0)	50 (100.0)	73 (100.0)
>60 days	99 (80.5)	37 (74.0)	62 (84.9)
>120 days	91 (74.0)	33 (66.0)	58 (79.5)
>180 days	81 (65.9)	29 (58.0)	52 (71.2)
>240 days	80 (65.0)	29 (58.0)	51 (69.9)
>300 days	76 (61.8)	28 (56.0)	48 (65.8)
>360 days	75 (61.0)	28 (56.0)	47 (64.4)
≥365 days (censored)	75 (61.0)	28 (56.0)	47 (64.4)

MPR: medication possession ratio; SD: standard deviation; TTD: time to discontinuation.

^a^Early initiators started treatment during months 1–4 of once-daily teriparatide availability, whereas later initiators started treatment during months 5–8 of once-daily teriparatide availability.

^b^The sum of the days' supply of medication dispensed between the start and the end of the study period divided by the total number of days (365) in the study period.

^c^Time from the start of treatment to the discontinuation of treatment. Discontinuation was defined as a 60-day gap in once-daily teriparatide supply.
